# Context Matters: Factors Affecting Implementation of Simulation Training in Nursing and Midwifery Schools in North America, Africa and Asia

**DOI:** 10.1016/j.ecns.2022.10.004

**Published:** 2023-02

**Authors:** Jami Baayd, Zoë Heins, Dilys Walker, Patience Afulani, Mona Sterling, Jessica N. Sanders, Susanna Cohen

**Affiliations:** a ASCENT Center for Sexual and Reproductive Health, Department of Obstetrics and Gynecology, University of Utah School of Medicine, Salt Lake City, UT, USA; b Global Medical Affairs Scientist, bioMerieux, Salt Lake City, UT, USA; c School of Medicine, Institute for Global Health Sciences, University of California, San Francisco, CA, USA; d Department of Epidemiology and Biostatistics and Institute for Global Health Sciences, University of California, San Francisco, CA, USA; e Lumina Alliance, San Luis Obispo, CA, USA; f ASCENT Center for Sexual and Reproductive Health and LIFT Simulation Design Lab, Department of Obstetrics and Gynecology, University of Utah School of Medicine, Salt Lake City, UT, USA

**Keywords:** simulation, nursing, midwifery, education, low-resource, CFIR, implementation

## Abstract

**Background:**

Robust evidence supports the effectiveness of simulation training in nursing and midwifery education. Simulation allows trainees to apply newly-learned skills in a supportive environment.

**Method:**

This study was conducted using the Consolidated Framework for Implementation Research (CFIR). We conducted in-depth individual interviews with simulation experts around the world.

**Results:**

Findings from this study highlight best-practices in facilitating simulation implementation across resources settings. Universal accelerators included: (1) adaptability of simulation (2) “simulation champions” (3) involving key stakeholders and (4) culturally-informed, pre-implementation planning.

**Conclusions:**

Shared constructs reported in diverse settings provide lessons to implementing evidence-based, flexible simulation trainings in pre-service curriculum.

## Highlights

• Simulation training can be adapted to meet students' needs in a range of settings.
• culturally-informed planning is crucial when introducing simulation into new settings.
• Key stakeholder should be engaged early in implementation process of new program.

**Key Points**

• High-quality simulation education is not dependent on the type of equipment or the style of the simulation lab, but on the commitment and excitement of faculty and students.
• Successful implementation of simulation programs in low-resource settings requires efforts by implementers to understand the unique attributes of specific settings, and adapt simulation to fit the realities of the culture and context of the learner.
• Involving key stakeholders early on in the process of implementing new simulation training programs improved the implementation process and achieved better results.

## Introduction

Opportunities to practice skills in clinical settings is a crucial component of nursing and midwifery education. (Davis et al., 2018; Marzalik, Feltham, Jefferson, & Pekin, 2018; Panda, Dash, & John, 2021) Limited clinical opportunities during training can create competency gaps for newly-graduated nurses. (Michel, Ryan, & Mattheus, 2021; Modarres, Geranmayeh, Amini, & Toosi, 2022; Ochylski, Aebersold, & Kuebric, 2017) An increasing number of schools worldwide are turning to simulation to increase opportunities for hands-on experiences. (Fuglsang, Bloch, & Selberg, 2022; Mulyadi, Tonapa, Rompas, Wang, & Lee, 2021)

Robust evidence supports the effectiveness of simulation training across medical education including in nursing and midwifery. (Doğru & Aydın, 2020; Kuo, Wu, Chen, Chen, & Hu, 2020; Labrague, McEnroe-Petitte, Bowling, Nwafor, & Tsaras, 2019; Stoodley, McKellar, Steen, & Fleet, 2020; Tamaki et al., 2019; Tarrahi, Kianpour, Ghasemi, & Mohamadirizi, 2022) Simulation provides trainees an opportunity to apply their newly learned skills and receive real-time feedback in a supportive environment. (Aebersold, 2018; Lateef, 2020; Roh, Jang, & Issenberg, 2021) Though simulation, students can learn how to manage rare and high-risk medical scenarios. (Dahlberg, Nelson, Dahlgren, & Blomberg, 2018; Ghosh, Cohen, & Spindler, 2022; Nielsen, Nikolajsen, Paltved, & Aagaard, 2021) Increasing frequency of exposure to rare and high-risk events in simulated environments allow for students develop habits of best-practices for handling emergencies. (Kelly, Berragan, Husebo, & Orr, 2016) Participation in pre-service simulation training is also associated with improved assessment skills, (Ochylski et al., 2017) better teamwork and communication, (Davis et al., 2018; Kelly et al., 2016) improved clinical and academic performance by students, (Al Gharibi, Koukab, & Arulappan, 2020) and an increased ability to assess and prioritize needs of a patient. (Ochylski et al., 2017)

Simulation has become standard practice in nursing and midwifery schools in high-resource settings due to this demonstrated effectiveness. (Smiley, 2019) While less common, evidence also supports use of simulation training in low-resource settings. (Alfes & Madigan, 2017; Mehdipour–Rabori, Bagherian, & Nematollahi, 2021; Najjuma et al., 2020; Tjoflåt, Våga, & Søreide, 2017) Most reports on pre-service simulation trainings focus on the Western standard of care and educational practices at Western institutions, with little information about using the methodology for institutions in other parts of the globe. (Martinerie, Rasoaherinomenjanahary, & Ronot, 2018) Identifying best-practices and common pitfalls across diverse settings, including geographical, cultural, language and available resources, will allow for standard guidance on how to introduce and integrate simulation trainings into existing education curriculum. Additionally, allowing for these approaches to be both evidence-based and flexible will support greater impact globally. This study aimed to understand common barriers and facilitators to introducing simulation training to nursing and midwifery schools across low/mid- and high- resource settings.

## Methods

### Theoretical Framework

This study was designed, conducted and analyzed using the Consolidated Framework for Implementation Research (CFIR). (Research CRT-CfCM 2019) The CFIR was developed to guide systematic analysis of an organization's readiness for implementing new programs or interventions. The CFIR has been used to assess the implementation process in a variety of settings. (Chan et al., 2021; Li, Benbow, & Keiser, 2022; van Oers, Teela, Schepers, Grootenhuis, & Haverman, 2021; Ware et al., 2018) We used the CFIR to retrospectively examine implementation of simulation in each location, and to understand what slowed the process (decelerators) and what sped up the process (accelerators) of implementing. Prior to conducting the interviews, our team of researchers and simulation content experts reviewed the CFIR constructs and selected the constructs that applied to simulation. The relevant constructs were then used to develop the interview guide.

### Data Collection

For this qualitative study, we conducted in-depth individual interviews with simulation experts from nursing and midwifery programs around the world. We started with purposive sampling based on known simulation experts identified in a literature review. We then relied on snowball sampling to invite experienced simulationists from diverse settings, representing a range of geographic regions, cultural backgrounds, and high, mid and low-resource settings. Individuals were invited to participate if they had direct involvement in the introduction or implementation of simulation into nursing or midwifery education. Interviews were conducted by the first author, in English. All authors have participated in simulation design and research, the last author for over 15 years. Interviews were conducted in-person (n = 1) or remotely via Skype or telephone (n = 13), using a semi-structured interview guide. Interviews were audio recorded and transcribed verbatim. All participants provided verbal consent prior to the interviews. Interviews took between 30 minutes and 120 minutes and participants were not compensated for their time. The study was approved by Institutional Review Boards from the University of Utah and University of California, San Francisco.

### Analysis

The interview transcripts were analyzed according the process outlined by the CFIR. (Research CRT-CfCM 2019) First, interview transcripts were coded according to a modified CFIR codebook, using the qualitative data analysis software, NVivo 12 Plus. (NVivo 2018) A portion of the transcripts were double coded to measure inter-rater reliability, which was determined to be over 90 percent. Once an entire transcript was coded, we grouped each of the coded quotes by construct. Those construct clusters were then separately scored by two members of the analysis team (JB and ZH) based on whether the construct was a decelerator or an accelerator to adopting simulation in the setting in which the interviewee worked (the scale was -2, -1, 0, Mixed, +1, +2). If there was discordance between the two coders, we discussed the construct and representative quotes, and reached consensus on the rating. After each construct was scored for each transcript, the construct scores for all transcripts were aggregated, and each construct was given an overall rating (representing the aggregate construct score across all transcripts). This final rating was determined through consensus between the two coders and the process was then repeated for only high-resource settings and only low/mid-resource settings. Classification as a low and middle resource, or high-resource setting, was based on World Bank country income classification. (Group TWB June 2018)

We use descriptive and comparative analysis to better understand meaningful differences between the accelerators and decelerators to implementing simulation in various settings and examine differences in construct ratings between high, low and middle resource settings.

## Results

Our study team interviewed 14 people. Participants were simulationists from a variety of medical backgrounds, with both clinical and administrative roles represented. Eleven of the participants had been trained in simulation in the USA or Canada, and nine were then invited to implement their simulation program in another country. During the interviews, we asked participants to focus on the country where they had the most extensive simulation experience, as many of the participants had introduced simulation programs in several countries. Four participants focused on their experience in high-resource countries, and nine focused on their experience implementing in low-resource countries. Table 1 details the location, school type and professional role of the interviewees.

**Table 1:** Simulation Programs Represented in Study

Simulation Program Locations	
India	3
United States	4
South Sudan	1
Qatar	1
Canada	1
Tanzania	1
Namibia	1
China	1
East Asia*	1
Student population served by simulation program†	
Nursing students	11
Midwifery students	9
Medical Students	5
Professional background of simulation experts	
Nurse-midwife educator	6
Nurse educator	7
Medical doctor/educator	1

⁎ Interviewer asked to keep country anonymous

† Most programs serve multiple types of students and are therefore represented more than once

The analysis yielded codes for 32 total constructs. For this study, we will report on the results of the 14 constructs which were present in at least seven different (or half of the sample) interview transcripts. See Table 2 for a definition of the 14 constructs, all of which are modified definitions of constructs from the CFIR codebook.

**Table 2:** CFIR Constructs, Modified for Simulation Context

Significant Constructs
Adaptability The degree to which simulation can be adapted, tailored, refined, or reinvented to meet student's needs and the program curriculum.
Available Resources The level of resources dedicated to the implementation and on-going operation of a simulation program.
Organizational Culture Norms, values, and basic assumptions of a given organization. Culture of an organization may mirror culture of the external setting.
Design Quality Perceived excellence in how simulation program and materials are bundled, presented, and assembled.
Engaging Champions Attracting and involving early adopters in the implementation and use of simulation to overcome indifference/resistance to simulation.
Engaging Key Stakeholders Attracting and involving individuals within the organization and outside of the organization who have a vested interest in simulation.
Engaging Students Attracting and involving students who will participate in simulation.
External Policy External strategies that directly facilitated or hindered the implementation of simulation at an institution.
Faculty Engagement Commitment, involvement, and accountability of faculty with the implementation of simulation.
Innovation Source Perception of key stakeholders about whether the simulation is externally or internally developed.
Knowledge & Beliefs Individuals’ attitudes toward and the value placed on simulation, as well as familiarity with facts and principles related to simulation.
Planning The degree to which tasks for implementing simulation are developed in advance, and the quality of those schemes or methods.
Structural Characteristics The social architecture, age, maturity, and size of the organization/university where the simulation program was introduced.
Student Needs The extent to which the needs of students are met using simulation.

Figure (below) shows each of the constructs’ aggregate rating (negative rating indicates a decelarator or barrier, and positive rating indicates an accelarator or facilitator). The size of the circle indicates the number of interview transcripts in which the construct was present.

**Figure 1:**
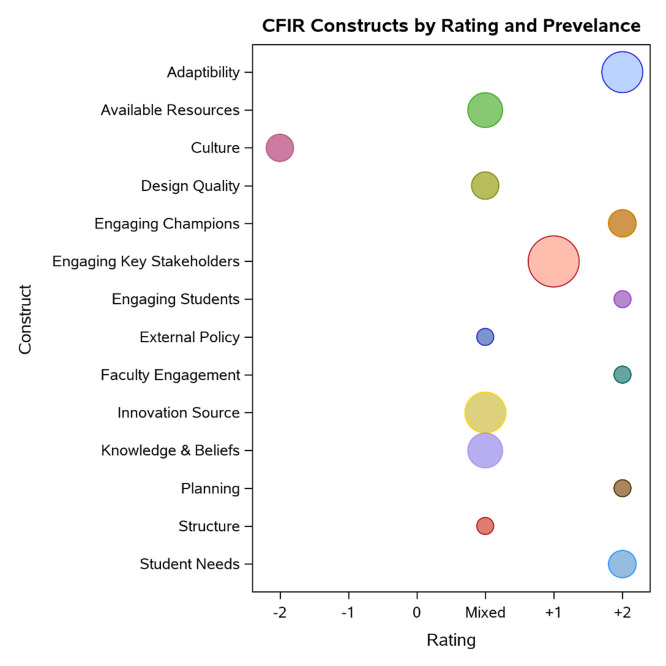
CFIR constructs by rating and prevelance.

As evidenced in the ratings, *culture (organizational culture)* was the primary decelerator in implementation. Many of the constructs had mixed ratings with *available resources, innovation source*, and *knowledge and beliefs about simulation* being the most commonly discussed during interviews and highlighted as both barriers and facilitators to effective implementation. *Engaging key stakeholders,* the *adaptability of simulation* and the ability of simulation to meet *student needs* were the most common accelerators to implementation. Select constructs are described in more detail, below.

## Culture/Organizational Culture

Each of the implementing institutations had organizational cultures which reflected the broader societal culture they were sitatuted within. The cultural disconnect between Western-style simulation learning theory and pedagogy in implementation countries was the most universally mentioned decelerator to implementing simulation. Although the inherent adaptability of simulation as a teaching methodology (see *adaptability* construct) prevented this difference in culture from completely stalling implementation at most institutions, time and effort were required to effectively mold simulation practices to unique student bodies. Interviewees stressed that the burden should be on implementers to adapt simulation training to the cultural context. This means implementers must understand how to adapt simulation training to fit not only the clinical, but also the cultural, needs of their students.

“In educational systems that don't teach or value critical thinking skills, simulation isn't just a new methodology, it is a whole new way of thinking. This makes simulation potentially incredibly impactful but also slows the implementation process considerably.” –Namibia

“Hierarchies within the culture make it difficult to implement simulation both in the acting out of scenarios and in the feedback following the simulation.” –India

“Simulation implementers must put in the effort to adapt simulation to fit the realities of the culture. If we [the educators] don't change simulation to fit the realities of the culture, we have failed (not the simulation).”

–United States

## Available Resources

Interestingly, financial resources were not the most significant predictor of the availability of simulation resources. Some programs with relatively little funding invested wisely, growing their programs by allocating resources for faculty development, and providing physical and educational support for simulation. Other programs with considerable disposable income spent most of their funds purchasing expensive equipment, or large simulation labs, but did not invest in training the necessary staff to effectively run a simulation program.

Additionally, at institutions where most of the simulation materials were donated, the donated materials, including simulation mannequins, were often not suitable for the setting. The donated mannequins sometimes required specific training to be run properly or were incompatible with the electrical current at the institution, making them unusable.

“They're a resource-rich environment in terms of physical resource, so they've got money, they've got buildings, they've got equipment, but they didn't have the people with the requisite backgrounds in teaching and health education” –Qatar

“Somebody donated one of those [high-tech mannequins] but I don't think she's intact because anything that needs to be plugged in becomes quite problematic, so I think she broke quite early in the game. The electrical current changes so everything gets fried.”–South Sudan

## Innovation Source

Many of the simulation experts we interviewed were from the US or Canada and were invited to begin new simulation programs in other countries. In some settings, stakeholders felt that having the innovation sourced outside of the country was advantageous because it provided access to outside resources and expertise. Stakeholders in other settings felt that because the innovation was externally sourced, participants were reticent to engage with what they viewed as a foreign intervention. In those situations, implementation success was achieved by pairing with a trusted individual from within the organization. In countries where the innovation source was internal (i.e., where the implementer originated from the country where the program was implemented) was found to be a universal accelerator to implementation.

And helping [administrators] understand that [simulation] is not about the technology, that mistakes are made in the sim lab and not in clinical practice makes them comfortable, but you've got to have a faculty ally. You know, you're always an outsider. I don't even care if you're visiting faculty for five years, you're an outsider. So there has to be a faculty ally, there has to be somebody who buys into it.-United States

## Knowledge and Beliefs About Simulation

When stakeholders at an institution had previous exposure to high-quality simulation, it was often the driving force that brought the idea of simulation to the institution in the first place. Unfortunately, some individuals had exposure to poor quality simulation or something that was called simulation but actually was not. These experiences soured them on the idea of simulation and made it more difficult to garner their support for the implementation of simulation at their own facilities. Additionally, the misconception that expensive simulators are necessary for a successful simulation program hindered implementation at some institutions with fewer financial resources.

“Sometimes there are people who no matter what, they hate it [simulation]. On the whole, if somebody will give themselves the chance and just go in and sit down and watch one in their discipline all the way through, they tend to absolutely see how wonderful it could be.”–United States

“In high and low resource countries people imagine high tech robotic models [when they think of simulation]. It's unfortunate that the term sim is getting misinterpreted. I see people from low-resource settings and people dismiss it.”–Southeast Asia

## Engaging Key Stakeholders

Key stakeholders involved with the implementation of simulation in nursing programs included individuals from within institutions– such as faculty, staff, and administration– as well as key stakeholders from outside of the institution– government officials, health departments, and investors. Regardless of who the stakeholders were, interviewees reported best results when the stakeholders were involved early on in the process of implementation. Implementers were most successful in engaging stakeholders when they demonstrated a simulation for them. Some interviewees stated that stakeholders responded well when simulation scenarios were highly complex, while others found that faculty were anxious when participating in difficult simulations.

Although the process of engaging various stakeholders can be time-consuming, buy-in, especially from leadership, seems to be essential for implementation to be successful. Without the support of decision-makers the implementation process will halt.

“Involving faculty, health departments, institutional leaders and administration early and often will ease implementation but may be time-consuming.”– Namibia

“They started to make mistakes, and then it felt more real. Then suddenly people are like, “oh my god, this is so great.” Then once again, when the leadership didn't like it, we just did a double simulation, and then when they saw that it was bringing in so much reality, and they were like, “oh so this works.” So, the ones who have more knowledge, they always need a little more challenge to be convinced that simulation can make good effect.”–India

## Adaptability of Simulation

One of the biggest strengths in implementing simulation training is that scenarios can be adapted to individual learning environments, and the simulation materials and simulators available. Interviewees specifically discussed the ability of simulation to adapt to different religious requirements, learning styles, class sizes, educational levels, and working environments.

“You just have to look at the system and decide how to create a team out of the resources that are there.”–Namibia

“And I think just asking them that question; ‘this is what we would do, but I am sensing that maybe this is not what you would do. How could you adapt this, how could we adapt this to be appropriate for your culture?’”–United States

## Meets Needs of Students

Those we interviewed shared that almost universally, students were hungry for new learning opportunities and chances to practice their skills in a realistic setting. Students found simulation to be an exciting change from didactic learning. In many places, a shortage of clinical developmental opportunities makes simulation particularly important. The excitement of the students was an accelerator to implementing simulation, as students would often advocate for implementation thus encouraging more reluctant faculty or administration.

“The students don't get enough hands-on training… but what we could offer her [nursing student] is to come to the sim lab and get trained. . .she would love to work in the hospital but our policies [don't allow it] … but we are able to expose her to the sim training and she is able to appreciate that.” –India

“We really found that nurses wanted to be more empowered and they wanted to be able to practice to their full scope. They also wanted to be able to use technology and be empowered to use that technology when they got to the hospital.” –India

## Design Quality

For this study, we considered *design quality* to include the design of the simulation scenarios and the type of simulation technology used (standardized patient, mannequin, standardized patient wearing or using a simulation device). Design quality was variable for simulation programs—in some instances well designed simulations and functioning simulation technology led to smooth implementation as students and stakeholders saw the best of what simulation could offer. In other cases, the simulation technology was not well-suited for the simulation learning objectives, or programs did not have the resources or training to use the technology appropriately. In those cases, the design quality was a clear decelerator to implementation. This can be a particular decelerator to implementation in low/mid-resource countries where schools often do not have the resources to maintain the high-tech equipment initially purchased, and the simulation program becomes defunct as a result. The primary difference between simulation technology being an accelerator or decelerator was whether the technology was purchased to suit the program objectives, or if the technology was purchased first, and then the program objectives were built around the technology.

“I just don't think that the actual tools are the focus of the sim, I think it's really the communication and the people getting up and moving and doing and learning through using their bodies. I'm a really strong believer in this whole, physical literacy thing, where you physically do stuff while you're saying it out loud while working with people, it actually opens up your neural pathways to remember stuff. So, I just actually don't really necessarily-low tech, high tech, whatever,-I actually don't think it makes a difference.” -South Sudan

“You have to design. It's part of your design. A simulation is not a scenario. A simulation is an educational event that's starts with a proper needs assessment, knowing your population, knowing what they need to learn, where are the gaps that you're filling in your curriculum. You know, proper educational design. I'll tell you what's happening in these [other simulation] programs, now I don't have any stats on this but I can tell you anecdotally that this is what's happening, is that people are closing their simulation programs. They say, ‘sorry but you know what, guess what, we have no faculty to run this, we invested heavily in all this really expensive equipment.”-Qatar

Table 3 describes the results for the analysis by country income classification. A majority of constructs (13 of 24) were rated as strong accelerators in high-resource countries, while a smaller number, (7 of 25) were rated as such in low/mid-resource countries. Conversely, in low/mid-resource settings, a majority of constructs (12 of 25) fell under the rating of “mixed,” meaning that respondents’ experiences at different institutions were too incongruent to give the construct either a positive or negative rating.

**Table 3:** A Heat Map Comparison of Decelerators (red) and Accelerators (orange) in High versus Low Resource Settings

## Discussion

Findings from this study highlight best-practices in facilitating simulation implementation across settings and offer demonstrated approaches to overcoming key barriers. Some accelerators to implementing simulation were consistent across both high- and low- resources settings, suggesting universal prerequisites to starting and maintaining simulation training programs. These universal accelerators were (1) the adaptability of simulation to fit the needs of a pre-service program, (2) identification and involvement of “simulation champions,” (3) early identification and involvement of key stakeholders (school administration, deans and faculty, government officials, health departments and funders) and (4) high quality pre-implementation planning. While organizational culture was identified as a universal decelerator.

The construct with the most substantial discrepancy between high-resource and low-middle resource countries was *External Policy*. For high-resource countries, this construct was a strong accelerator. Governments tended to be supportive of simulation programs and even contributed to the programs financially. In contrast, in low-resource settings, this construct was rated as a strong-decelerator. In these settings, implementers found that rigid, nationally-mandated curriculums for nursing schools made it difficult to introduce new content.

Another construct which showed considerable disagreement between high-resource and low-resource countries is *Design Quality*. Although implementers in both high and low/mid-resource settings stressed that simulators do not need to be high-tech to deliver high-quality learning experiences, the idea that expensive simulators were necessary for a successful simulation program persisted.

There are several limitations to this study. The small sample size and the use of a non-random sampling approach limit generalizability to all simulation education training. Most of the respondents were individuals from high-resource settings who entered as non-nationals to implement simulation in low/mid-resource settings. Thus, the views presented for low/mid-resource settings represent the opinions of outsiders trying to implement simulation in a new setting. Additionally, we were not able to recruit participants from Europe, Australia or South America thereby limiting the diversity of perspectives. All the interviews were conducted in English, restricting opportunities for non-English speaking participants. The study also has several strengths. First, it is among the first studies to assess accelerators and decelerators to introducing simulation into pre-service programs across various settings. Additionally, the study is strengthened by the use of the CFIR, a well-established implementation science framework.

## Conclusion

The incorporation of simulation into pre-service curriculum has many benefits to the learners as well as improving outcomes in the future. Those who implement simulation can use findings from this study to ensure that best-practices are incorporated into any new simulation curriculum. These best-practices lie not in the type of equipment used or the style of the simulation lab, but in the commitment and excitement of faculty and students. Successful implementation of simulation programs in low/mid-resource settings requires efforts by implementers to understand the unique attributes of specific settings and to adapt simulation to fit the realities of the culture and context of the learner. Additionally, it is important to engage key stakeholders early in the process and to recruit, train, and support faculty simulation champions. Furthermore, introducing students to simulation early on will help the new program gain traction and acceptance. Additional research and evaluation on simulation training in nursing and midwifery education will create opportunities to build on these best practices and expand the potential impact of this dynamic educational tool.

## Declaration of Competing Interest

The authors have no conflicts of interest to disclose.
